# Perineural invasion as a prognostic factor for intrahepatic cholangiocarcinoma after curative resection and a potential indication for postoperative chemotherapy: a retrospective cohort study

**DOI:** 10.1186/s12885-020-06781-w

**Published:** 2020-03-30

**Authors:** Zeyu Zhang, Yufan Zhou, Kuan Hu, Dong Wang, Zhiming Wang, Yun Huang

**Affiliations:** Department of Hepatobiliary Surgery, Xiangya Hospital, Central South University, Changsha, Hunan China

**Keywords:** Intrahepatic cholangiocarcinoma, Perineural invasion, Postoperative chemotherapy, Curative resection, Survival

## Abstract

**Background:**

In the past four decades, the incidence of cholangiocarcinoma, especially intrahepatic cholangiocarcinoma (ICC), has raised rapidly worldwide. Completeness of resection, max size of tumor and etc. are widely recognized as prognostic factors. However, the prognosis significance of perineural invasion (PNI) on recurrence-free survival (RFS) and overall survival (OS) in ICC patients is controversial.

**Methods:**

ICC patients who underwent curative hepatectomy and diagnosed pathologically were retrospectively analyzed. Patients were grouped by existence of PNI and outcomes were compared between groups. The potential relationship between PNI and postoperative chemotherapy was also investigated.

**Results:**

There was no significant difference in demographic, clinical staging or tumor index between two groups, except positive hepatitis B surface antigen and CA19–9. PNI negative group showed a better prognosis in RFS (*P* < 0.0001) and OS (*P* < 0.0001). COX regression analyses showed PNI as an independent risk factor in RFS and OS. ICC with postoperative chemotherapy showed better effects in the whole cohort on both RFS (*P =* 0.0023) and OS (*P* = 0.0011). In PNI negative group, postoperative chemotherapy also showed significant benefits on RFS and OS, however not in PNI positive group (*P* = 0.4920 in RFS and *P* = 0.8004 in OS).

**Conclusion:**

PNI was an independent risk factor in R0-resected ICC, presenting worse recurrence and survival outcomes. Meanwhile, negative PNI may act as an indication of postoperative chemotherapy.

## Background

In the past four decades, the incidence of cholangiocarcinoma (CCA), which is now the second most common hepatic malignancy following hepatocellular carcinoma (HCC), has raised rapidly worldwide [1–5]. CCA is derived from bile duct epithelium and usually grows aggressively without symptoms until advanced stage. Meanwhile, unlike HCC, diagnosing CCA at an early stage and treating at an advanced stage remain challenges, eventually causing the poor prognosis of the patients with CCA [6–8].

CCA can be divided into 2 main groups: extrahepatic (ECC) including hilar type and distal type, and intrahepatic (ICC) including peripheral type and hilar type based on the location of tumor. It has been increasingly identified that there are distinct epidemiologic, clinical and biologic characteristics between ICC and ECC [9], so they are usually studied separately. In another classification system of ICC based on tumor morphology, ICC can be subdivided into 3 types: mass forming, periductal infiltrating, and intraductal growth [10]. But the differences of clinical characteristics and outcome between these types are still controversial.

As well as HCC, treatments toward ICC are limited [11, 12]. For patients with advanced-stage or unresectable ICC, locoregional and chemotherapeutics are the primary treatment options, while surgery is the main treatment for resectable ICC and provides a potential curative method [13]. However, even after complete resection, overall survival is unsatisfactory in ICC (5–43%) [14, 15]. In addition, as a potential beneficial adjuvant therapy after surgery, the role of postoperative chemotherapy is still unclear. A few of clinical data and meta analysis reveal its positive effect on clinical outcomes in ICC patients, but currently no randomized clinical trial supports it [16]. Moreover, the indication for postoperative chemotherapy is also unknown.

Perineural invasion (PNI), as tumor cell invasion though perineurium, is one of the widely studied pathologic factors in various malignant tumors [17–20]. Different from metastasis which is via the bloodstream or lymphatic system, PNI is a process with distinctive histologic features, underlying cellular mechanisms, and molecular mediators [21]. Although the definition of PNI is still controversial [22, 23], the significance of PNI as a risk factor representing a poor prognosis in ECC is well shown [24, 25]. However, the prognosis significance of PNI on recurrence-free survival (RFS) and overall survival (OS) in ICC patients is controversial. The aims of this study are to determine the effect of PNI on prognosis in R0-resected ICC patient and to clarify the potential relationship between PNI and postoperative chemotherapy.

## Methods

### Study population

Patients who underwent curative hepatectomy and pathologically diagnosed as ICC at the Xiangya Hospital of Central South University between January 2012 and December 2016 were enrolled for the selection of patients. The inclusion criteria of the selection included: 1) aged 18–75 years old; 2) newly diagnosed ICC without any previous anti-tumor treatment; (3) underwent curative hepatectomy with negative surgery margin (R0 resection); (4) mass forming type of ICC.

Patients who did not undergo curative hepatectomy (R1 and R2) were excluded. ECC patients were excluded as the center of the tumor was below the bifurcation of the common hepatic duct according to the 8th AJCC (American Joint Committee on Cancer) Cancer Staging Manual. Cases would be excluded when the origin of tumor was hard to distinguish. In addition, the origin of a periductal infiltrating or an intraductal growth type of ICC could be hard to distinguish. So only mass forming type of ICC was enrolled in this study.

### Data collection and follow up

The medical histories and pathology reports were reviewed for basic information, clinical data and tumor characteristics. Patients will be divided into two groups (PNI positive group and PNI negative group) according to their situation of PNI. The TNM stage was evaluated using the 8th AJCC Cancer Staging Manual. In the T stage classfication, vascular invasion contained both macrovascular invasion and mircovascular invasion. And in our medical center, lymphadenectomy was not regularly performed in patients without enlarged lymph nodes detected by imaging examination or intraoperative exploration. The N stage of patient who did not received lymphadenectomy by any reasons was evaluated as Nx.

The main outcomes were recurrence-free survival (RFS) and overall survival (OS). RFS time and OS time were calculated from the time of surgery. Follow-up was completed on January 15, 2019. The study was approved by the ethics committee of Xiangya Hospital of Central South University (no. 2018121140). Patient consent was not required to review their medical records by the ethics committee of Xiangya Hospital of Central South University because of its retrospective design, and exemption from informed consent did not adversely affect the health and rights of subjects. This study kept confidentiality of patient data and strictly complied with the Declaration of Helsinki and its later amendments or comparable ethical standards.

### Statistical analysis

Statistical Package for Social Sciences 22.0 was used for all the statistical analyses. The continuous variables were expressed as mean ± standard deviation or median value (range) and analyzed by using independent-sample t test or Mann-Whitney U test as appropriate. Categorical variables were expressed as frequency (percentage) and analyzed using Chi-square or Fisher exact test as appropriate. Kaplan-Meier (K-M) curves was used for survival analyses, and log-rank test was applied to analyze differences between groups. Univariate and multivariate Cox proportional hazard regression were applied to identify significant risk factors of survival data. Factors with *P* < 0.10 in univariate analysis were included in multivariate analysis where the method of Forward: LR was used. All statistical assessments were two-tailed, and *P* < 0.05 was considered statistically significant.

## Results

### Patient and tumor characteristics

A total of 134 patients were enrolled in this study, 76 patients were presented as PNI negative while 58 as PNI positive. Clinicopathologic characteristics of two groups were comparatively shown in Table 1. Most of ICC were peripheral type in both group (*P* = 0.273), and 38 patients presented multiple tumors (*P* = 0.548). According to the 8th AJCC Cancer Staging Manual, 47 patients were divided as T1, while 20 as T2, 41 as T3, 26 as T4 (*P* = 0.486). Lymph node (LN) metastasis was pathologically confirmed in 32 patients (*P* = 0.503). 34 patients were defined as stage I, 19 as stage II, and 81 as stage III (*P* = 0.672). Overall, there was no statistically significant difference in tumor characteristics between groups.

**Table 1:** Clinicopathologic characteristics

Characteristic	PNI negative (*n* = 76)	PNI positive (*n* = 58)	*P*
Age (years)	57.16 ± 10.28	55.34 ± 8.24	0.273
Male	41 (53.9)	35 (60.3)	0.459
Location			0.781
Hilar	13 (17.1)	11 (19.0)	
Peripheral	63 (82.9)	47 (81.0)	
HBsAg			0.001
Negative	46 (60.5)	50 (86.2)	
Positive	30 (39.5)	8 (13.8)	
Tumor size (cm)			0.945
≤ 5	31 (40.8)	24 (41.4)	
> 5	45 (59.2)	34 (58.6)	
Multiple tumor			0.548
No	56 (73.7)	40 (69.0)	
Yes	20 (26.3)	18 (31.0)	
Vascular invasion			0.148
No	50 (65.8)	31 (53.4)	
Yes	26 (34.2)	27 (46.6)	
Capsular invasion			0.521
No	39 (51.3)	33 (56.9)	
Yes	37 (48.7)	25 (43.1)	
Visceral invasion			0.441
No	63 (82.9)	45 (77.6)	
Yes	13 (17.1)	13 (22.4)	
AJCC T stage			0.486
1 and 2	36 (47.4)	31 (53.4)	
3 and 4	40 (52.6)	27 (46.6)	
AJCC N stage			0.503
0	52 (68.4)	34 (58.6)	
1	16 (21.1)	16 (27.6)	
x	8 (10.5)	8 (13.8)	
AJCC tumor stage			0.672
I	20 (26.3)	14 (24.1)	
II	9 (11.8)	10 (17.2)	
III	47 (61.8)	34 (58.6)	
Tumor differentiation			0.133
Well to moderate	29 (38.2)	15 (25.9)	
Poor to undifferentiated	47 (61.8)	43 (74.1)	
Liver cirrhosis			0.071
No	45 (59.2)	43 (74.1)	
Yes	31 (40.8)	15 (25.9)	
ALT (U/L)			0.116
≤ 40	52 (68.4)	32 (55.2)	
> 40	24 (31.6)	26 (44.8)	
AST (U/L)			0.656
≤ 40	50 (65.8)	36 (62.1)	
> 40	26 (34.2)	22 (37.9)	
PLT (×10^9/L)	225.55 ± 92.82	231.22 ± 91.17	0.725
CEA (ng/ml)			0.910
≤ 5	57 (75.0)	43 (74.1)	
> 5	19 (25.0)	15 (25.9)	
CA19–9 (U/ml)			0.017
≤ 200	51 (67.1)	27 (46.6)	
> 200	25 (32.9)	31 (53.4)	
CA242 (U/ml)			0.134
≤ 20	44 (57.9)	26 (44.8)	
> 20	32 (42.1)	32 (55.2)	
Child-Pugh score			0.259
A	68 (89.5)	48 (82.8)	
B	8 (10.5)	10 (17.2)	
Postoperative chemotherapy			0.409
No	66 (86.8)	53 (91.4)	
Yes	10 (13.2)	5 (8.6)	
Post-recurrence anti-tumor therapy			0.987
Yes	51 (67.1)	5 (67.2)	
No	25 (32.9)	28 (32.8)	

Data are expressed as mean ± standard deviation or n (%)

*PNI* perineural invasion, *HBsAg* hepatitis B surface antigen, *AJCC* American Joint Committee on Cancer, *ALT* Alanine aminotransferase, *AST* Aspartate aminotransferase, *PLT* Blood platelet, *CEA* Carcinoembryonic antigen;

As for clinical features, no statistical significance was detected in liver cirrhosis, alanine aminotransferase (ALT), aspartate aminotransferase (AST), blood platelet (PLT), carcinoembryonic antigen (CEA), CA24–2 and Child-Pugh score. Particularly, positive hepatitis B surface antigen (HBsAg) was shown in 30 patients (39.5%) in PNI negative group while 8 patients (13.8%) in PNI positive group (*P* = 0.001). The difference in CA19–9 level between two groups was also considered as statistically significant (*P* = 0.017), revealing higher CA19–9 level in PNI positive group.

### Survival analysis

**Fig. 1:**
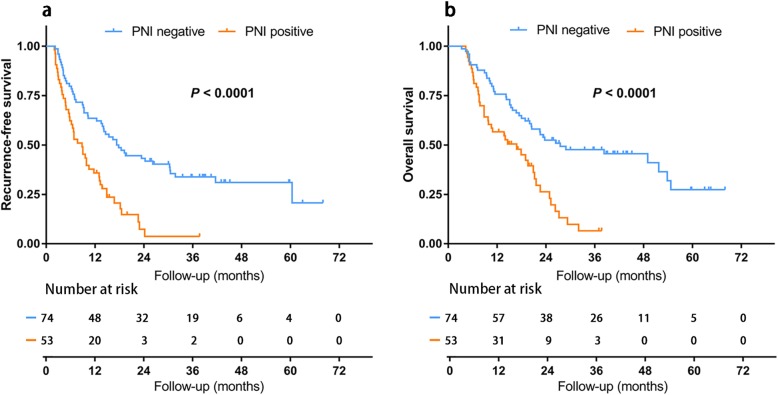
Comparison of RFS (**a**) and OS (**b**) in patients with and without PNI. PNI, perineural invasion; RFS, recurrence free survival; OS, overall survival

7 patients who died from severe postoperative complications were removed from survival analysis. Among the remaining 127 patients, the survival analysis of RFS and OS was performed between groups with the results shown in Fig. 1. The medium follow up time was 18.0 months. At the time of last follow-up, 49 (66.2%) patients with negative PNI and 44 (83.0%) with positive PNI suffered from tumor recurrence. 43 (58.1%) patients with negative PNI and 40 (75.5%) with positive PNI suffered from death. The median RFS and OS were 17.30 months (95% CI: 12.14–22.46) and 27.50 months (95% CI: 6.10–48.91) in patients with negative PNI, while 8.80 months (95% CI: 5.85–11.76) and 16.80 months (95% CI: 9.01–24.59) in patients with positive PNI. RFS rates for patients with negative PNI were 63.5% at 1 year, 33.9% at 3 years, while 35.8% at 1 year, 3.7% at 3 years for patients with positive PNI. OS rates for patients with negative PNI were 75.7% at 1 year, 47.6% at 3 years, while 56.6% at 1 year, 6.6% at 3 years for patients with positive PNI. Log-rank test showed significant differences between two groups in both RFS (*P <* 0.0001) and OS (*P <* 0.0001), which meant better prognoses of patients with negative PNI in both RFS and OS.

Univariate and multivariate Cox proportional hazard regression of RFS and OS were performed among the 127 patients and results were shown in Tables 2 and 3, respectively. Large size of tumor, multiple tumors, positive PNI, lymph node metastasis, low tumor differentiation, liver cirrhosis, high level of CA19–9 and lack of postoperative chemotherapy could make a worse effect on RFS. As for OS, absence of PNI, low AJCC stage, high tumor differentiation, low level of preoperative AST and CA19–9, postoperative chemotherapy were beneficial.

**Table 2:** Univariate and multivariate analysis for recurrence-free survival

Variable	Reference	Univariate COX regression		Multivariate COX regression	
		HR (95% CI)	*P*	HR (95% CI)	*P*
Age	< 60	0.743 (0.492, 1.121)	0.156		
Gender	Female	0.894 (0.598, 1.335)	0.894		
Location	Hilar	1.186 (0.701, 2.004)	0.525		
HBsAg	Negative	0.909 (0.585, 1.412)	0.671		
Tumor size (cm)	≤5	1.913 (1.250, 2.928)	0.003	1.834 (1.152, 2.920)	0.011
Multiple tumor	No	1.699 (1.100, 2.625)	0.017	1.701 (1.060, 2.731)	0.028
Vascular invasion	Negative	1.473 (0.982, 2.210)	0.061	–	0.138
Perineural invasion	Negative	2.504 (1.634, 3.836)	0.000	2.562 (1.564, 4.199)	0.000
Capsular invasion	Negative	1.345 (0.900, 2.010)	0.148		
Visceral invasion	Negative	1.416 (0.855, 2.345)	0.177		
AJCC T stage	T1 and T2	1.305 (0.874, 1.949)	0.193		
AJCC N stage	N0 and Nx	2.449 (1.542, 3.891)	0.000	1.742 (1.050, 2.891)	0.032
AJCC stage	I				
Stage II		2.784 (1.415, 5.477)	0.003	–	0.633
Stage III		2.333 (1.378, 3.950)	0.002	–	0.266
Tumor differentiation	Well to moderate	2.140 (1.366, 3.352)	0.001	2.796 (1.718, 4.550)	0.000
Liver cirrhosis	Negative	1.463 (0.971, 2.204)	0.069	1.992 (1.264, 3.139)	0.003
ALT (U/L)	≤40	1.074 (0.710, 1.624)	0.737		
AST (U/L)	≤40	1.158 (0.763, 1.757)	0.490		
PLT (×10^9/L)	≤300	1.411 (0.874, 2.276)	0.159		
CEA (ng/ml)	≤5	1.698 (1.085, 2.657)	0.020	–	0.737
CA19–9 (U/ml)	≤200	2.784 (1.841, 4.209)	0.000	2.625 (1.624, 4.243)	0.000
CA24–2 (U/ml)	≤20	2.147 (1.426, 3.232)	0.000	–	0.568
Child-Pugh classification	A	1.016 (0.575, 1.794)	0.957		
Postoperative chemotherapy	No	0.296 (0.129, 0.680)	0.004	0.282 (0.119, 0.668)	0.004

*HR* hazard ratio, *CI* confidence interval, *PNI* perineural invasion, *HBsAg* hepatitis B surface antigen, *AJCC* American Joint Committee on Cancer, *ALT* Alanine aminotransferase, *AST* Aspartate aminotransferase, *PLT* Blood platelet, *CEA* Carcinoembryonic antigen

**Table 3:** Univariate and multivariate analysis for overall survival

Variable	Reference	Univariate COX regression		Multivariate COX regression	
		HR (95% CI)	*P*	HR (95% CI)	*P*
Age	< 60	0.861 (0.555, 1.335)	0.503		
Gender	Female	0.709 (0.461, 1.091)	0.118		
Location	Hilar	1.138 (0.648, 1.996)	0.653		
HBsAg	Negative	0.817 (0.508, 1.315)	0.406		
Tumor size (cm)	≤5	1.798 (1.135, 2.849)	0.012	–	0.115
Multiple tumor	No	1.804 (1.136, 2.867)	0.012	–	0.551
Vascular invasion	Negative	1.297 (0.838, 2.006)	0.243		
Perineural invasion	Negative	2.515 (1.592, 3.975)	0.000	1.747 (1.080, 2.826)	0.023
Capsular invasion	Negative	1.391 (0.905, 2.140)	0.133		
Visceral invasion	Negative	1.646 (0.971, 2.790)	0.064	–	0.718
AJCC T stage	T1 and T2	1.332 (0.865, 2.050)	0.193		
AJCC N stage	N0 and Nx	2.805 (1.717, 4.585)	0.000	–	0.331
AJCC stage	I				
Stage II		4.876(2.239, 10.617)	0.000	3.307 (1.466, 7.460)	0.004
Stage III		3.473 (1.814, 6.647)	0.000	2.799 (1.444, 5.424)	0.002
Tumor differentiation	Well to moderate	1.923 (1.179, 3.139)	0.009	2.179 (1.309, 3.626)	0.003
Liver cirrhosis	Negative	1.297 (0.837, 2.011)	0.245		
ALT (U/L)	≤40	1.272 (0.816, 1.982)	0.289		
AST (U/L)	≤40	1.463 (0.942, 2.274)	0.091	1.612 (1.003, 2.590)	0.049
PLT (×10^9/L)	≤300	1.834 (1.122, 2.997)	0.016	–	0.156
CEA (ng/ml)	≤5	1.936 (1.213, 3.092)	0.006	–	0.114
CA19–9 (U/ml)	≤200	3.220 (2.057, 5.042)	0.000	2.911 (1.798, 4.712)	0.000
CA24–2 (U/ml)	≤20	2.427 (1.554, 3.790)	0.000	–	0.983
Child-Pugh classification	A	1.355 (0.762, 2.411)	0.301		
Postoperative chemotherapy	No	0.180 (0.057, 0.573)	0.004	0.174 (0.054, 0.566)	0.004
Post-recurrence anti-tumor therapy	No	0.871 (0.557, 1.361)	0.543		

*HR* hazard ratio, *CI* confidence interval, *PNI* perineural invasion, *HBsAg* hepatitis B surface antigen, *AJCC* American Joint Committee on Cancer, *ALT* Alanine aminotransferase, *AST* Aspartate aminotransferase, *PLT* Blood platelet, *CEA* Carcinoembryonic antigen

**Fig. 2:**
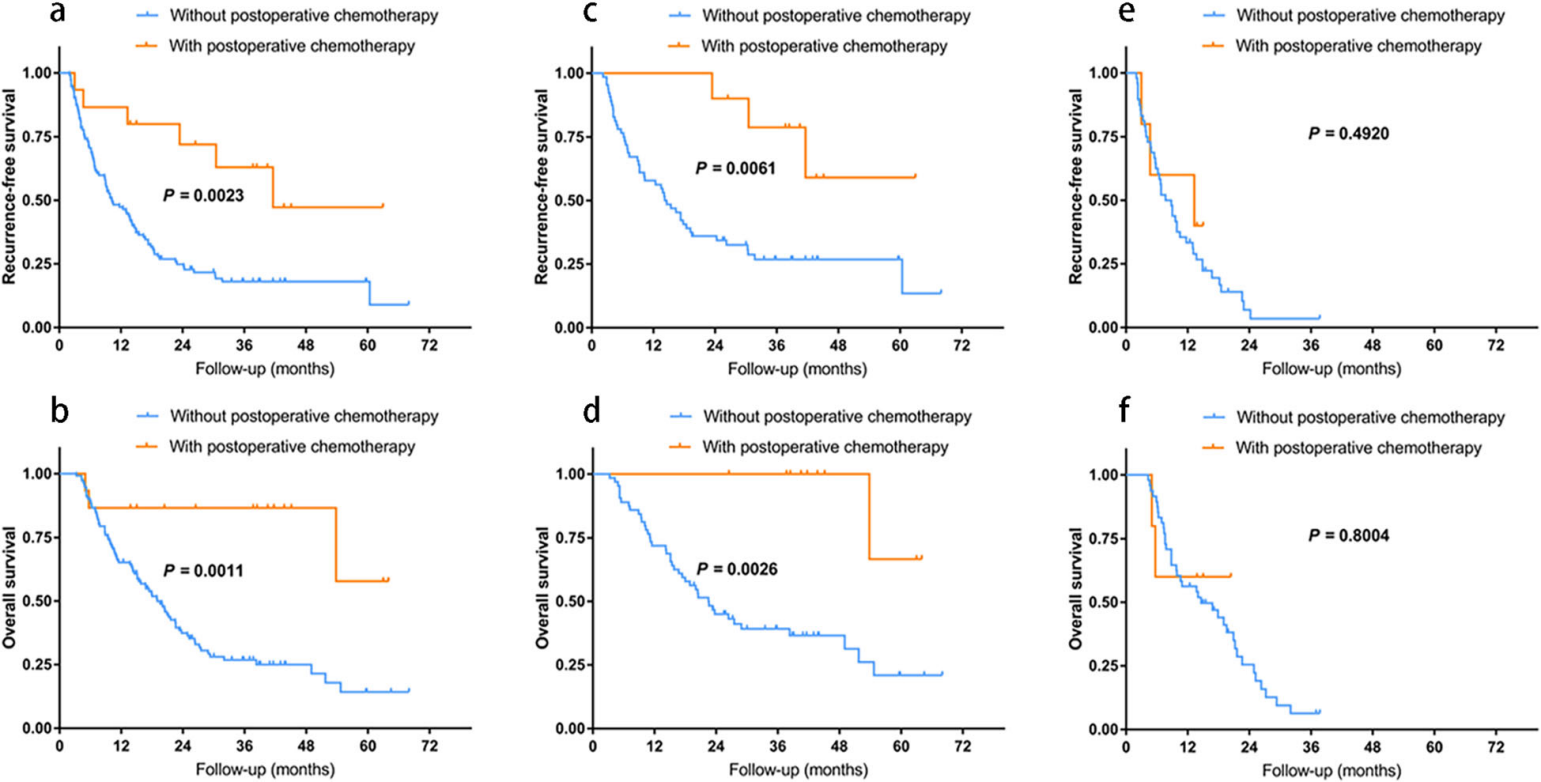
Postoperative chemotherapy showed significant survival improvement on RFS (**a**) and OS (**b**) in the whole cohort of patients, and on RFS (**c**) and OS (**d**) in the patients without PNI. However, it did not showed any improvement on RFS (**e**) or OS (**f**) in the patients with PNI. PNI, perineural invasion; RFS, recurrence free survival; OS, overall survival

Further analyses were performed to illustrate potential relationship between PNI and postoperative chemotherapy, and the details of postoperative chemotherapy were showed in Table 4. After regrouping 127 patients into with postoperative chemotherapy group and without postoperative chemotherapy group, there was no significant difference in any clinicopathological factors between two groups (Table 5). Using K-M curve, postoperative chemotherapy made significant benefits on both RFS (*P =* 0.0023) and OS (*P* = 0.0011) among the whole 127 patients (Fig. 2a and b). Moreover, among the 76 patients with negative PNI (Fig. 2c and d), postoperative chemotherapy also showed as beneficial to both RFS (*P* = 0.0061) and OS (*P* = 0.0026). However, among the 58 patients with positive PNI (Fig. 2e and f), postoperative chemotherapy did not prolong RFS (*P* = 0.4920) or OS (*P* = 0.8004).

**Table 4:** Details of postoperative chemotherapy

	Patients, n (%)
Capecitabine (1250 mg/m^2^ twice daily on days 1–14 of a 3-week cycle)	3 (20.0%)
Gemcitabine (1000 mg/m^2^ on days 1, 8 and 15 of a 4-week cycle)	2 (13.3%)
Gemcitabine + Cisplatin (1250 mg/m^2^ + 30 mg/m^2^ on days 1 and 8 of a 3-week cycle)	9 (60.0%)
Gemcitabine + Capecitabine (1000 mg/m^2^ on days 1 and 8 + 1250 mg/m^2^ twice daily on days 1–14 of a 3-week cycle)	1 (6.7%)

**Table 5:** Clinicopathologic characteristics

Characteristic	Without postoperative chemotherapy (*n* = 112)	With postoperative chemotherapy (*n* = 15)	*P*
Age (years)	56.19 ± 9.79	55.13 ± 7.94	0.690
Male	59 (52.7)	10 (66.7)	0.307
Location			0.203
Hilar	18 (16.1)	5 (33.3)	
Peripheral	94 (83.9)	10 (66.7)	
HBsAg			0.494
Negative	81 (72.3)	9 (60.0)	
Positive	31 (27.7)	6 (40.0)	
Tumor size (cm)			0.110
≤ 5	43 (38.4)	9 (60.0)	
> 5	69 (61.6)	6 (40.0)	
Multiple tumor			0.315
No	79 (70.5)	13 (86.7)	
Yes	33 (29.5)	2 (13.3)	
Vascular invasion			0.538
No	69 (61.6)	8 (53.3)	
Yes	43 (38.4)	7 (46.7)	
Capsular invasion			0.371
No	61 (54.5)	10 (66.7)	
Yes	51 (45.5)	5 (33.3)	
Visceral invasion			0.127
No	90 (80.4)	15 (100.0)	
Yes	22 (19.6)	0 (0.0)	
AJCC T stage			0.250
1 and 2	57 (50.9)	10 (66.7)	
3 and 4	55 (49.1)	5 (33.3)	
AJCC N stage			0.967
0	74 (66.1)	10 (66.7)	
1	25 (22.3)	3 (20.0)	
x	13 (11.6)	2 (13.3)	
AJCC tumor stage			0.364
I	28 (25.0)	6 (40.0)	
II	18 (16.1)	1 (6.7)	
III	66 (58.9)	8 (53.3)	
Tumor differentiation			0.643
Well to moderate	38 (33.9)	6 (40.0)	
Poor to undifferentiated	74 (66.1)	9 (60.0)	
Liver cirrhosis			0.450
No	71 (63.4)	11 (73.3)	
Yes	41 (36.6)	4 (26.7)	
ALT (U/L)			0.163
≤ 40	73 (65.2)	7 (46.7)	
> 40	39 (34.8)	8 (53.3)	
AST (U/L)			0.297
≤ 40	75 (67.0)	8 (53.3)	
> 40	37 (33.0)	7 (46.7)	
PLT (×10^9/L)	232.32 ± 94.43	208.60 ± 87.96	0.359
CEA (ng/ml)			0.859
≤ 5	83 (74.1)	12 (80.0)	
> 5	29 (25.9)	3 (20.0)	
CA19–9 (U/ml)			0.482
≤ 200	64 (57.1)	10 (66.7)	
> 200	48 (42.9)	5 (33.3)	
CA242 (U/ml)			0.910
≤ 20	58 (51.8)	8 (53.3)	
> 20	54 (48.2)	7 (46.7)	
Child-Pugh score			0.228
A	99 (88.4)	11 (73.3)	
B	13 (11.6)	4 (26.7)	
Perineural invasion			0.482
No	64 (57.1)	10 (66.7)	
Yes	48 (42.9)	5 (33.3)	
Post-recurrence anti-tumor therapy			0.489
Yes	72 (64.3)	11 (73.3)	
No	40 (35.7)	4 (26.7)	

Data are expressed as mean ± standard deviation or n (%)

*PNI* perineural invasion, *HBsAg* hepatitis B surface antigen, *AJCC* American Joint Committee on Cancer, *ALT* Alanine aminotransferase, *AST* Aspartate aminotransferase, *PLT* Blood platelet, *CEA* Carcinoembryonic antigen

## Discussion

This study was performed among the ICC patients who underwent curative hepatectomy. The results of the present study revealed that patient with positive PNI had a worse prognosis in both RFS and OS. Besides, postoperative chemotherapy could significantly prolong both RFS and OS, especially in patients with negative PNI. However, with limited patient number, it seemed no benefit to apply postoperative chemotherapy in patient with positive PNI. In a word, our study suggested PNI as a significant prognostic factor in ICC and postoperative chemotherapy may benefit ICC patients who went underwent curative resection, especially patients with negative PNI.

PNI has been recognized and studied as a prognostic factor for decades in various tumor. However, studies of PNI toward ICC are comparatively fewer, and the results are controversial. The significance of PNI affecting OS in ICC patients was reported at early days in 2000s [26–28]. In recent studies, several studies also reported the same results. Fisher et al. [29] retrospectively analyzed 58 ICC patients (36 with negative PNI and 22 with positive PNI) and revealed the patients with positive PNI had worse OS regardless of situation of LN metastasis. Ahn et al. [30] retrospectively analyzed 292 R0-resected ICC patients and the univariate and multivariate survival analyses of OS showed PNI as an independent significant risk factor against long-term survival, which is consistent with our results. However, some studies revealed quite different results that did not support PNI as one [31, 32]. Comparatively speaking, fewer studies report the meaning of PNI to RFS. As far as we concerned, only studies from Kang et al. [31] and Chan et al. [32] showed PNI had no influence in RFS through the univariate and multivariate analyses in their case-control studies, which are not consistent with our results. However, we did go further by using study design of historical cohort, K-M curves and the univariate and multivariate analyses to make a more convincible evidence. Nevertheless, PNI is not currently considered as an independently significant risk factor in the 8th AJCC Cancer Staging Manual. Based on our results, we consider large scale researches and a meta analysis are worth doing to determine the prognostic effect of PNI, thus may provide stronger evidences for putting PNI into the cancer staging system.

The situation of postoperative chemotherapy in ICC is still debating. So far, few studies reported the effect of chemotherapy on ICC patients with R0 resection. And no randomized phase III clinical trial data demonstrated a significant survival advantage in ICC from postoperative chemotherapy and none of clinical guidelines strongly recommended it. This was the very reason that we did not recommend postoperative chemotherapy to patients with resectable ICC, which caused small number of patients underwent chemotherapy after R0 resection in this study. Kim et al. [33] demonstrated that chemotherapy was not associated with a survival advantage in R0-resected ICC. Similarly, a meta-analysis including 19 studies showed that postoperative chemotherapy could improve OS and survival in patients with R1 resection in CCA, but did not benefit patients with R0 resection [34]. However, our results showed a significant benefit on both RFS and OS in patients with R0 resection. On the other hand, the indication of postoperative chemotherapy is also controversial [16]. Horgan et al. [35] observed that adjuvant chemotherapy was associated with improved survival among CCA patients with LN metastasis. However, the study contained few ICC patients. In the present study, although with a small cohort, we observed a great survival improvement through postoperative chemotherapy in patients with negative PNI and preliminary identify negative PNI as a possible indication of postoperative chemotherapy. But we still believed more studies should be performed on this issue in future before applying.

In the comparison of clinicopathologic characteristics, positive PNI was associated with negative HBsAg (*P* = 0.001), which meant ICC patients with hepatitis B virus (HBV) infection would be less likely to have PNI. Interestingly, the same phenomenon appeared in other studies (7 of 37 ICC patients in HBV group and 93 of 255 ICC patients in non-HBV group had PNI, *P* = 0.036 [30]; *P* = 0.009 in a meta-analysis [36]), which may indicate potential associations between HBV infection and genesis of PNI. Moreover, HBV infection is considered as a predictor of favorable survival outcomes for ICC [36], which is explained by early discovery during regular examination for HBV infection. However, it may also be explained by negative PNI according to our results. On the other hand, it is known that HCC and ICC have a common carcinogenic disease process if HBV infection is present [37, 38], revealing different carcinogenic disease processes between HBV group and non-HBV group in ICC. Thus we consider the causality and mechanism between HBV infection and PNI in ICC are worth to be studied in future. In addition, HBV infection may be associated with indication of postoperative chemotherapy as well if HBV infection is somehow connected with PNI.

The first limitation was that our study included a relatively small number of patients which might reduce our ability to demonstrate the results of our present study. Especially, a small number of patients underwent postoperative were included because of the reason we discussed above. Considering unusualness of ICC, a multi-center study with a large mount of patient is required in the future. Another limitation of our study was its retrospective design with unavoidable bias. A prospective randomized phase III clinical trials should be performed to provide higher grade evidences for significance of PNI as a prognostic factor and clearly determine the role of postoperative chemotherapy for R0-resected ICC patients with or without PNI. In addition, patient characteristics showed PNI positive group with higher CA19–9 level which was revealed to be an independent risk factor in RFS and OS according to results of Cox regression analysis, which has been widely recognized and included in ICC cancer staging system. However, we considered the prognostic effect of PNI was independent from CA19–9 level, since multivariate Cox proportional hazard regression included both CA19–9 and PNI. Lastly, our study was fail to demonstrate the difference between various regimen of chemotherapy and there was no randomized phase III clinical trial data to support a standard chemotherapy regimen. As for the regimens in the present study, chemotherapy for biliary tract cancers has traditionally followed the regimens used for advanced pancreatic cancers including gemcitabine, capecitabine, cisplatin, oxaliplatin, and carboplatin [39]. And national comprehensive cancer network (NCCN) clinical practice guidelines in oncology (version 1.2018) showed the similar suggestions. Future works should also be performed toward this issue.

## Conclusion

We observed that R0-resected ICC patients with PNI showed worse recurrence and survival outcomes comparing to patients without PNI, indicating PNI as a significant prognostic factor in ICC. In the meantime, negative PNI may act as an indication of postoperative chemotherapy. Randomized controlled trials should be performed to provided stronger evidences.

## Data Availability

All data generated or analyzed during this study are included in this published article.

## Abbreviations

ICC: Intrahepatic cholangiocarcinoma
PNI: Perineural invasion
RFS: Recurrence-free survival
OS: Overall survival
CCA: Cholangiocarcinoma
HCC: Hepatocellular carcinoma
ECC: Extrahepatic cholangiocarcinoma
AJCC: American Joint Committee on Cancer
K-M curves: Kaplan-Meier curves
LN: Lymph node
ALT: Alanine aminotransferase
AST: Aspartate aminotransferase
PLT: Platelet
CEA: Carcinoembryonic antigen
HBsAg: Hepatitis B surface antigen
HBV: Hepatitis B virus
NCCN: National Comprehensive Cancer Network
